# Pathology and bacteria related to digital dermatitis in dairy cattle in all year round grazing system in Brazil

**DOI:** 10.1371/journal.pone.0193870

**Published:** 2018-03-07

**Authors:** Tiago F. Moreira, Elias J. Facury Filho, Antônio U. Carvalho, Mikael L. Strube, Martin W. Nielsen, Kirstine Klitgaard, Tim K. Jensen

**Affiliations:** 1 Department of Veterinary Clinics and Surgery, School of Veterinary Medicine, Federal University of Minas Gerais, Belo Horizonte, Minas Gerais, Brazil; 2 National Veterinary Institute, Technical University of Denmark, Copenhagen, Denmark; Wageningen UR Livestock Research, NETHERLANDS

## Abstract

Digital dermatitis (DD) is one of the main causes of lameness in dairy cattle worldwide, and it is frequently reported in high-yielding, free stall dairy herds from regions with a temperate climate. However, DD is also observed with high prevalence in grazing cattle with a low milk yield in tropical regions. To clarify whether these differences have an impact on the etiology of the disease, we studied DD lesions from all year round grazing cattle of mixed breed in Brazil using high-throughput 16S rRNA gene sequencing and fluorescent *in situ* hybridization. The study included samples from 66 skin lesions and 5 healthy skins collected from five farms. Both techniques showed *Treponema* spp. to be the most abundant bacteria, present in all but one of the samples with minimal epidermal alterations. We identified eleven different *Treponema* strains belonging to the six major phylotypes of *Treponema* which have all previously been identified in DD lesions. Furthermore, we identify *Dichelobacter nodosus* in DD lesions by gene sequencing and also by fluorescent *in situ* hybridization in almost half of biopsy specimens in areas with mild epithelial damage and together with *Treponema*. The present data support the hypothesis that *Treponema* constitutes the main pathogen responsible for DD, independent of the environment and region where cows are kept, and it further suggests *D*. *nodosus* as another potentially important pathogen.

## Introduction

Since first being reported in 1974, bovine digital dermatitis (DD) has spread all over the world to reach an endemic situation in several countries [1, 2]. In the last decades, it has become a growing issue in dairy cattle and presently represents the main cause of lameness in cattle [1–3]. DD is an inflammatory skin lesion characterized by ulceration and/or papillomatous growths, usually located on the plantar surface of the hind foot and normally circumscribing the heel bulbs [2]. Lesions are painful and may result in lameness, reduced milk yields, impaired reproductive performance and premature culling [4]. The severe consequences of this disease makes it a great concern for the dairy industry worldwide and a subject of much attention [4].

DD is a polymicrobial disease for which the etiology still remains to be elucidated [5]. Spirochetes of the genus *Treponema* are the most prevalent bacteria in the lesions, and they are normally found in the deep part of the lesions in the layer between damaged and healthy tissue [3, 6, 7]. To date, more than 20 different *Treponema* phylotypes have been identified from DD lesions and among these, *Treponema phagedenis-like*, *Treponema refringens-like Treponema medium*/ *vincentii-like*, *Treponema denticola- like*, and *Treponema pedis* were the most prevalent. Other bacteria which have been identified from DD lesions are *Mycoplasma*, *Fusobacterium*, *Porphyromonas* [5, 6] *Bacteroides* spp., *Campylobacter* spp., *Guggenheimella* spp [7, 8] and *D*. *nodosus* [9], but the role played by each of these bacterial groups is not well known. As many *Treponema* spp. are not yet cultivable, culture independent methods, such as next generation sequencing (NGS) and fluorescent *in situ* hybridization (FISH), have been used with success to study DD etiology [6, 10].

The first report of DD in Brazil was in 1992, by Borges et al. (1992) [11]. Since then, several authors have reported DD with prevalence ranging from 0.92% [12] to 44.2% [13]. A recent study using nested PCR identified three *Treponema* phylogroups in DD lesions in Brazil, *T*. *medium*/*T*. *vincentii*-like, *T*. *phagedenis*-like, and *T*. *denticola*/*T*. *putidum*-like, but no details about the housing were provide [14]. Previous studies of digital dermatitis have focused on samples from dairy herds with Holstein cows using free stalls in temperate climates [1, 15–17]. However, despite the great differences in environmental and management conditions, DD is also observed with high prevalence in grazing cattle [13]. These differences may have an impact on the epidemiology and the etiology of the disease. In this context, the aim of this study was, therefore, to identify DD-associated pathogens from the lesions of all year round grazing cattle in Brazil in well-described housing systems, using high-throughput 16S rRNA gene sequencing and fluorescence *in situ* hybridization (FISH).

## Materials and methods

The set-up of this study was in accordance with the recommendations of The Animal Welfare Act of 1966 (AWA) (P.L. 89–544) and its amendments 1970 (P.L. 91–579); 1976 (P.L. 94–279), 1985 (P.L. 99–198). The project was previously approved by the Ethics Committee in Animal Experimentation from Universidade Federal de Minas Gerais (CEUA/UFMG) under protocol number 121 / 2015. All farm owners were in agreement with the study to be performed in their properties.

### Farms

Samples were collected from five different farms, all presenting a history of DD problems. Three farms is located in Prata (19° 18' 26" S 48° 55' 27" W), one in Martinho Campos (19° 19' 54" S 45° 14' 13" W) and one in Igarapé (20° 04' 13" S 44° 18' 06" W), all in Minas Gerais state, Brazil. The details of the farms are listed in Table 1. All farms kept the animals in pastures all year round and provided concentrate feed during milking or in feed pads after milking. Roughage supplementation with sugar cane and/or corn silage was provided during the dry period in all the farms. The daily milk production of the farms varied from 250L to 2000L and the average milk yield per cow from 9L to 20L per day. All animals on the farms were crossbred Holstein x Gyr cows. No preventive measures were taken regarding lameness and hoof lesions other than the use of a foot bath with copper sulfate or formalin solution in farms 4 and 5.

**Table 1:** Overview of characteristics of the 5 visited farms.

Characteristic	Farm 1	Farm 2	Farm 3	Farm 4	Farm 5
Lactating cows	100	28	56	83	86
Milk prod (L/day)	1200	250	900	1400	1600
Milk prod./cow (L)	12	9	16	16.9	19
DD prevalence (%)	15.8	41.2	36.6	42.3	46.5
Lameness prevalence (%)	18	31.4	42.5	12	35.1
Foot bath ^a^	-	-	-	+	+

^a^ Use (+) or no use (-) of foot bath in the farm

### Sample collection

DD lesions were cleaned thoroughly with water and dried off with paper towels, and biopsies were surgically excised after local anesthesia with lidocaine 2% subcutaneously or by bier block anesthesia. After this procedure, the surgical lesions were treated with topical tetracycline and bandages until the lesions were healed. The DD lesions sampled were classified according to Döpfer et al. (1997) [18] and adapted by Berry et al. (2012) [19]. Briefly, score M1 is a small active ulcer (<2 cm across) with moist surface and mottled red–gray coloring; M2 is a larger ulcerative active stage (>2 cm across) and normally painful upon manipulation; M3 is the healing stage where the lesion is covered by a dry brown scab, normally with no pain on manipulation; M4 is the chronic stage with proliferative hyperkeratotic growths that vary from papilliform to mass-like projections; M4.1 is the chronic stage with small active painful M1 focus.

The biopsies were subdivided, one half was placed in 10% neutral buffered formalin and then prepared for histological and FISH examination. The other half of the sample was stored in a nucleic acid stabilization solution (RNA*later*^*®*^, Ambion, Austin TX) for sequencing, and kept for 24 h at 5°C and then stored at -20°C until use.

### Fluorescent in situ hybridization (FISH)

For FISH analysis, serial sections of 4 μm were cut from the paraffin blocks and mounted on SuperFrost+ slides (Menzel-Gläser, Braunschweig, Germany). The oligonucleotide probes used in this study are listed in S1 Table and included probes specific for Domain Bacterium, *Treponema spp*., *F*. *necrophorum*, *D*. *nodosus*, *P*. *levii*, and nine *Treponema* phylotypes, *T*. *pedis*, *T*. *refringens*, *T*. *denticula*, *T*. *phagedenis*, *T*. *medium*, PT1, PT3, PT12, PT13. The probe for the domain Bacterium was used on all slides in combination with one other probe for individual bacterial species. The slides were mounted in a Sequenza slide rack (Thermo Shandon, Cheshire, United Kingdom) and kept for 14h in 45°C with 100μl of hybridization buffer (10 μl of 1 M Tris [pH 7.2], 18 μl of 5 M NaCl, 1 μl of 10% sodium dodecyl sulfate, 71 μl of H2O) with a concentration of 5 ng/μl of each applied oligonucleotide probe. The probe for the domain Bacterium was 5’ labeled with fluorescein isothiocyanate (FITC) and all other bacteria probes were 5’ labeled with the isothiocyanate derivative Cy3 (Eurofins MWG Operon, Ebersberg, Germany). The sections were then washed three times in pre-warmed (45°C) hybridization buffer for 5 minutes and subsequently washed three times in pre-warmed (45°C) washing buffer solution (10 ml of 1 M Tris [pH 7.2], 18 ml of 5 M NaCl, 72 ml of H2O) with the identical time interval. The sections were rinsed in water, air dried, and mounted in Vectashield (Vector Laboratories Inc., Burlingame, CA) for epifluorescence microscopy. An Axioimager M1 epifluorescence microscope equipped for epifluorescence with a 100-W HBO lamp and filter sets 43 and 38 was used to visualize Cy3 and FITC, respectively. Images were obtained using an AxioCam MRm version 3 FireWiremonocrome camera and AxioVision software, version 4.5 (Carl Zeiss, Oberkochen, Germany).

All biopsy specimens were scored from 0 to 3 according to the total bacteria invasion and the prevalence of each individual bacterial and *Treponema* phylotype, according to Nielsen et al. (2016) [6] and Rasmussen et al. (2012)[9]. Briefly, total bacteria invasion score 0 represents no invasive bacteria; score 1, low number of invasive bacteria; score 2, moderate number of invasive bacteria; score 3, high number of invasive bacteria. For individual bacteria score 0 signifies no bacteria; score 1, the specific bacteria represents up to 5% of the total number of bacteria; score 2, represents between 5% and 10% of the total number of bacteria; and score 3 represents more than 10% of the total number of bacteria.

### Histopathology

The slides used to perform FISH analysis were also used for histopathological evaluation afterwards. All biopsies were stained with hematoxylin and eosin (H&E). Epidermises were evaluated according to the presence and severity of acanthosis, parakeratosis, papillomatous proliferation, bacterial colonization showed by pale-staining and ballooning keratinocytes (swollen noneosinophilic cytoplasm and enlarged or condensed nuclei) and exocytosis, erosion, and/or ulceration of dermal papillae. Dermises were evaluated based on the inflammatory response present. Thereafter, the epidermal damage and inflammatory response in the dermis were scored from 0 to 3, adapted from Nielsen et al. (2016) [6]. Normal epidermis was defined as score 0, mild or focal epithelial proliferation and hyperkeratosis was defined as score 1, moderate epithelial proliferation and acanthosis was considered score 2 and extensive or diffuse damage with severe epithelial proliferation, acanthosis and exudation, erosion or necrosis of the dermal papilla was scored as 3. Inflammatory response was scored as 0 when there was no alteration, score 1 when there was a mild increase in the number of lymphocytes and mononuclear cells score 2 when the increase in inflammatory cells was moderate and score 3 when it was severe and diffuse. Scorings were based according to most severe alterations found in the biopsies. A Leica DMRB microscope was used for reading and images were obtained using a Leica MC120 HD camera and Leica Application Suite software, version 4.7.0 (Leica Microsystems, Wetzlar, Germany).

### DNA extraction

Bacterial DNA was extracted from lesions using a DNeasy tissue kit (Quiagen, Hilden, Germany). A 22 mg piece of fixed tissue was cut in small pieces with a scalpel. A sterile 5-mm steel bead (Quiagen, Hilden, Germany) was added, and samples were run two times on a TissueLyser II (Qiagen) at 20 Hz for 3 min per run in 180μl of ATL buffer. All the subsequent procedures were performed according to the protocols of the supplier. The concentrations and purity of the samples were evaluated using a Nanodrop 1000 spectrophotometer (Fisher Scientific, Wilmington, MA) and only samples with A260/A280 ratios of >1.8 were used for further analyses.

### Preparation of libraries and sequencing.

Amplification of DNA and library preparation was accomplished as described by Nielsen et al. (2016) [6]. Briefly, the DNA was amplified by PCR using a universal bacterial primer set which targets the V1–V2 region [20]. In order to determine the DD-associated spirochetes to the species level, we included a *Treponema*-specific primer set targeting the V3–V4 hyper variable regions of the 16S rRNA gene of the majority of treponemes hitherto identified in DD lesions [10]. The sequencing depth obtained with these *Treponema*-specific primers, enables better classification of this genus, even for low-abundant samples [10]. The prepared mixture for PCR contained 5 ml of 10xPCR Gold Buffer (Applied Biosystems, Foster City, CA, USA), 1.5 mM MgCl2 solution (Applied Biosystems), 200 mM of each deoxynucleoside triphosphate (Amersham Biosciences, Piscataway, NJ), 0.4 mM of each specific primer, 2.5 U of AmpliTaq Gold DNA polymerase (Applied Biosystems), and 2 ml of template DNA. The PCRs were performed in a T3 thermocycler (Biometram, Göttingen, Germany) following the steps of denaturation at 94°C for 6 min; 30 cycles of denaturation at 94°C for 45 s, annealing at 57°C for 45 s, extension at 72°C for 90s; elongation step of 10 min and cooling to 4°C. The primers had unique hexameric barcodes added at their 5’ ends to enable multiplexing.

DNA quality and concentration was assessed using an Agilent 2100 Bioanalyzer (Agilent Technologies Inc.). The resulted amplicons were pooled in equal amounts and purified with the Qiagen Mini Elute kit (Qiagen) according to the protocol. The DNA was submitted to the National High-Throughput DNA Sequencing Centre at the University of Copenhagen, Denmark for sequencing on the Illumina MiSeqTM platform. Sequences generated by Illumina MiSeq are available under the accession number PRJNA369034 in the NCBI Sequence Read Archive (SRA).

### Sequence analysis

Sequence analysis of 16S rRNA gene was performed by BION-meta software (http://box.com/bion). The de-multiplexing step was performed according to the primer and barcode sequences. Forward and reverse sequences were joined, allowing no gaps, a minimum match percentage of 80% and an overlap length of minimum 35 bp. Amplicons with qualities lower than 98%, which is equivalent to a Phred score of 17, were excluded. Sequences of at least 250 nucleotides in length were classified against the Ribosomal Database Project database II (RDP II; http://rdp.cme.msu.edu/index.jsp) using a word length of 8 and a match minimum of 90%. Then, the number of reads for each barcode was normalized for further analysis.

### Statistical analysis

Analysis was conducted using R software. To elucidate the microbial patterns involved in DD etiology, multivariate analysis of the resulting microbial profiles was carried out by Analysis of Similarities (ANOSIM) and comparison between control and DD samples regarding diversity of species were estimated using the Shannon index. A heatmap visualizing the Spearman correlations between majorly abundant families was generated in R.

## Results

In total, we evaluated 66 DD lesions and five control samples of healthy skin. The majority of the lesions were characterized macroscopically as being in classical active ulcerative stages. In 25.4% of the lesions the affected area was no more than 2cm across (M1) and in 43.3% of the lesions the affected area was >2cm across (M2). Healing stages (M3) represented 10.4% of the sampled lesions and were only found in farms with footbaths. The proliferative and wart-like conditions (M4) accounted for 20.9%. Three of the control samples were taken from healthy animals, whereas the last two control samples were taken from DD-affected animals, but from a healthy area of the hind feet 2 cm from the lesion area.

### Histopathology

**Fig 1:**
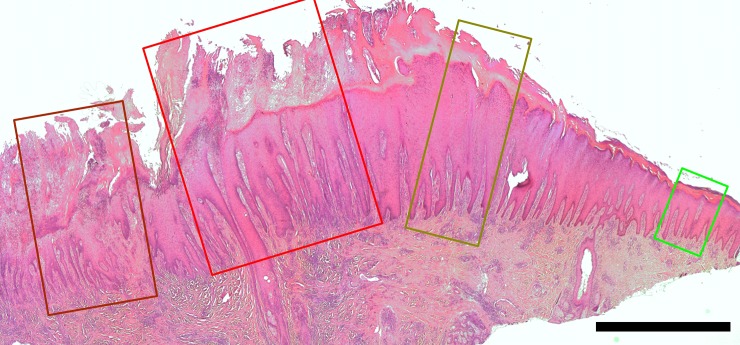
Skin biopsy from a dairy cow with ulcerative digital dermatitis macroscopically graded M2 according to Döpfer et al. (1997). Within the sample, different segments of the skin show typical epidermal and dermal alterations varying from left to right unaffected/healthy (green) score 0, moderate epithelial proliferation and acanthosis (score 2, dark green), extensive epidermal degeneration with severe epithelial proliferation, exudation and erosion (score 3, red). To the left, pointing to the center of the lesions, a score 3 segment with severe exudation and ulceration (dark red). H&E, bar 2mm.

Thirty-eight of the 66 DD biopsies, (57.6%) were characterized by severe epithelial proliferation, acanthosis, with some presenting papiliform projections. Furthermore, the epidermis had areas of erosion, ulcer, and the dermis presented exudation, necrosis of dermal papilla and extensive inflammatory infiltration (score 3). Twenty-six samples, (39.4%) presented a moderate increase in epidermis thickness without ulceration and moderate infiltration of inflammatory cells in the dermis (score 2), while the last two DD samples (3%) had only minor alterations manly represented by mild acanthosis. The healthy skin samples presented normal epidermises, besides the two control samples taken from affected feet, which presented mild hyperkeratosis (score 1). A biopsy revealing the different scoring stages of epidermal/dermal alterations from unaffected to ulcerative digital dermatitis, macroscopically graded M2, is shown in Fig 1.

### Fluorescent in situ hybridization FISH

**Fig 2:**
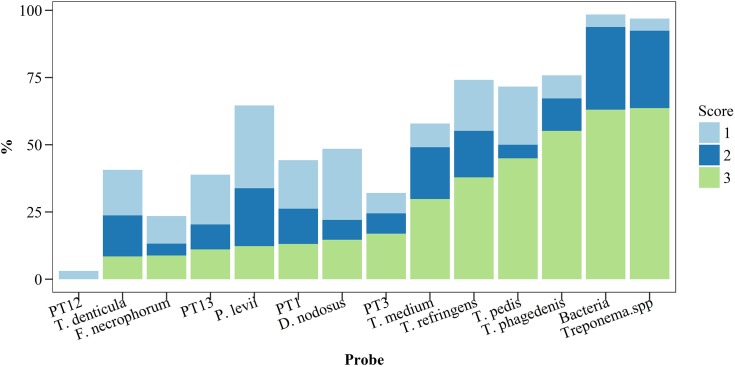
Prevalence and abundance score of different bacteria and *Treponema* phylotypes investigated by fluorescent *in situ* hybridization in biopsy specimens of bovine digital dermatitis (n = 66) from crossbreed dairy cows kept in pasture all year round. For total bacteria invasion score 1 represent a low number of invasive bacteria; score 2, moderate number of invasive bacteria; score 3, high number of invasive bacteria. For individual bacteria: score 1, bacteria represents up to 5% of the total number of bacteria; score 2, between 5% and 10% of the total number of bacteria; and score 3 represents more than 10% of the total number of bacteria.

We evaluated the 66 samples using double *in situ* hybridization targeting the domain Bacterium plus one species or genus specific probe, totalling 14 different probes. Fig 2 illustrates the prevalence and score for all bacteria and *Treponema* phylotypes investigated by FISH. Of the 66 samples, only one was negative for colonizing/invading bacteria, presenting only minimal epidermal alterations. *Treponema* spp. were the most frequent and abundant bacteria, present in 64 of the 66 (97%) samples. Furthermore, in 64% of the cases, treponemes represented the majority of the observed bacteria. *P*. *levii* was the second most prevalent bacteria with 64.6% positive samples, followed by *D*. *nodosus* (48.5%) and *F*. *necrophorum* (23.5%). Although very frequent, these three bacterial species represented less than 5% of the total bacteria present in most cases (Fig 2). Regarding the *Treponema* phylotypes, each lesion presented a median of 4 phylotypes and a maximum of 7 from the 9 tested phylotypes. The prevalences were, in decreasing order *T*. *phagedenis* (71%), *T*. *refringens* (68%), *T*. *pedis* (67.2%), *T*. *medium* (54.1%), PT1 (40.9%), PT13 (38.2%), *T*. *denticula* (37.5%), PT3 (30.4%), PT12 (3.3%). Two samples were negative for *Treponema* in FISH analysis. One of these was the sample negative for all tested probes. The other was classified as score 2 for dermal and epidermal damage and was positive *P*. *levii* in FISH. Additionally, *Treponema* were identified in this sample by sequencing analysis. One sample, which was taken from apparently healthy skin two cm from a lesion, was positive for *T*. *pedis*, *T*. *medium* and *T*. *phagadenis*. This sample had a score of 2 in the histopathological examination with altered dermis and epidermis and was likewise positive for *Treponema* in the NGS analyses.

**Fig 3:**
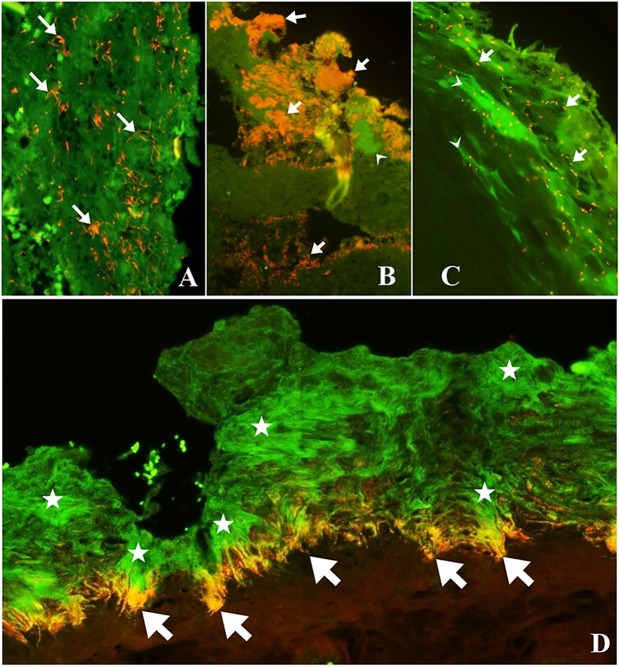
Fluorescent *in situ* hybridization on digital dermatitis biopsies demonstrating several bacteria. A) Fluorescent *in situ* hybridization demonstrating *Fusobacterium necrophorum* (red) (arrows) in small clusters and individual bacteria B) Double hybridization showing *Porphyromonas levii* (red) (arrows) organized in cluster in superficial layer and other bacteria in green (arrow head). C) Fluorescent *in situ* hybridization demonstrating *Dichelobacter nodosus* (red) (arrows) in sparse individual bacteria and other bacteria in green (arrow head). D) Digital dermatitis biopsy demonstrating massive and deep colonization of Spirochete-like bacteria (green) (stars) and *Treponema refringens* (red/orange) (arrows) in between damaged and healthy tissue.

Usually, spirochetes colonized the deeper parts of lesions with severe epidermal damage (score 3), forming a bright red fluorescing band between the healthy and affected tissue, as shown in Fig 3. Among the *Treponema* phylotypes, *T*. *pedis*, *T*. *medium*, *T*. *phagedenis*, and *T*. *refringens* were normally the ones present in the deeper parts of the lesions while *T*. *denticola*, and the uncultured *Treponema* PT1 were located superficially and PT12 were rarely found. *D*. *nodosus* were present as sparse single cells or as diplobacilli in areas of the skin with imperfect keratinization. Usually, *Treponema* and *D*. *nodosus* were the only bacteria present in areas where the epidermis and keratin layer where more preserved. *F*. *necrophorum* were present as single cells and as clusters, and normally together with other bacteria. *F*. *necrophorum* was only present in one sample without ulceration. *P*. *levii* formed small clusters in the very superficial layers of the lesions in distinguishing areas from *Treponema*. *F*. *necrophorum* and *P*. *levii* were found together occasionally once both bacteria were mainly present within ulcerated areas, with exudation or in more degradable epidermis.

Fig 1 illustrates the diverse histopathological aspects of the DD and the bacterial colonization pattern described previously. Compared to the bacterial abundance as revealed by FISH, invading bacteria were not observed in the green segment, whereas the superficial layers of the dark green segment commonly were invaded by varying numbers of *Dichelobacter nodosus* and treponemes. The red segments were characterized by a high number of invasive bacteria systematically with treponemes on the borderline between diseased and healthy tissue. *Fusobacterium necrophorum* and *Porphyromonas levii* were predominantly found in the ulceration of the dark red segment.

### 16S rRNA sequencing (NGS)

We successfully sequenced all 44 DD-lesions samples submitted and 4 of the 5 controls samples with the primers V1–V2 and 42 DD-lesions and 1 control sample with the primers V3–V4. The lower number of samples for sequencing compared to FISH is due to logistical matters and the availability of the nucleic acid stabilization solution. The reason some samples were not sequenced was that no PCR product could be amplified. The sequences were de-multiplexed according to the sequences of the barcodes and primers, and subsequently chimeras and sequences with quality below 98% were discarded. In total, 2,486,038 (V1–V2 pool) and 2,472,912 (V3–V4 pool) joined sequences were used for taxonomic classification. Of these sequences, 1,610,008 of the V1–V2 pool and 1,578,668 of the V3–V4 pool were taxonomically classifiable according to the RDPII database (http://rdp.cme.msu.edu/).

**Fig 4:**
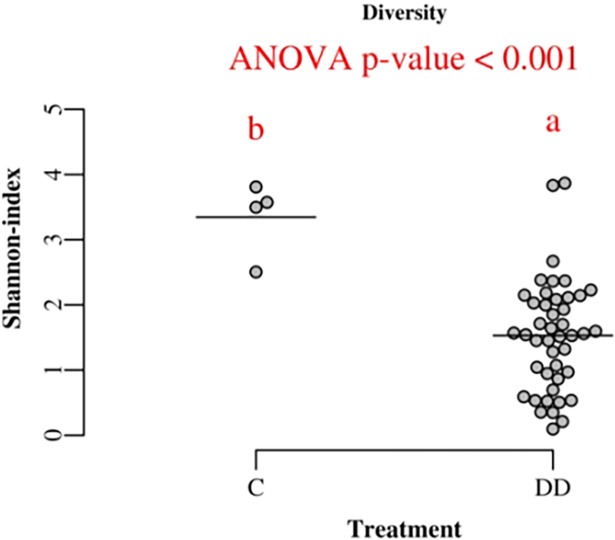
Diversity of bacterial genera in control and digital dermatitis specimens measured by Shannon-index.

The diversity of bacterial genera present in DD samples was lower than in the control samples (Fig 4). The number of different genera representing more than 1% of the total reads in DD samples was 23, while in controls this number was 35.

We were not able to detect any correlations between the bacterial species identified in NGS analysis and all the environmental parameters which might influence the composition of the DD microbiota, e.g.: macroscopic score of the lesion, use of footbath, sample region and farm.

**Fig 5:**
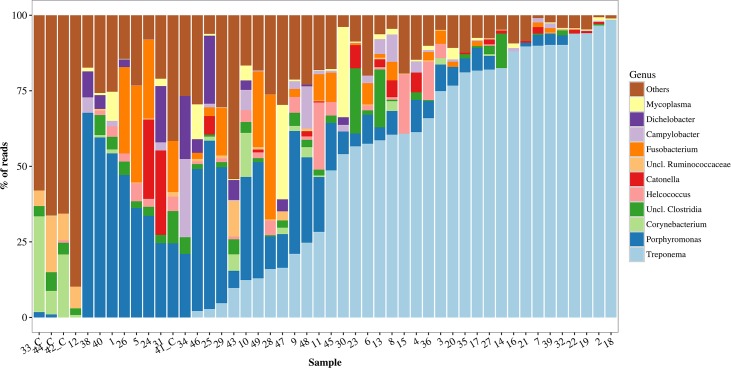
Relative abundance of bacterial genera amplified by high-throughput sequencing in bovine digital dermatitis. Sequencing of V1 and V2 hypervariable regions of 16S rRNA in 44 biopsy specimens of bovine digital dermatitis and 4 control samples from crossbreed dairy cows kept in pasture all year round. Control samples are identified by _C.

The most representative phyla represented in the sequencing results were in decreasing order *Spirochaetes*, *Bacteroidetes*, *Firmicutes* and *Fusobacteria*. The predominant genera in the DD samples were *Treponema* and *Porphyromonas* (Fig 5) with a prevalence of 86.36% for both genera. *Treponema* was the most abundant genus present in the DD lesions (Fig 5), representing more than 50% of total sequence reads in 52.3% of the samples with a mean abundance of 44.9%. *Porphyromonas* was the second most abundant bacteria and accounted for more than 20% of total reads in 31.8% of the samples with a mean abundance of 17.5%. Other genera that accounted for a high proportion of total reads were *Fusobacterium* (mean density of 5.15%), unclassified bacteria from class Clostridia (2.8%), *Helcococcu*s (2.5%), *Mycoplasma* (2.4%), *Dichelobacter* (2.1%), *Catonella* (2.1%) and *Campylobacter* (2.0%). Six DD samples were negative for *Treponema* in the results from V1-V2 primer. However, *Treponema* 16S RNA was successfully amplified by V3-V4 primer in four of these six samples, while in the other two samples *Treponema* was identified by FISH examination. In the six samples negative for *Treponema* in V1-V2 primer, *Porphyromonas* was the most abundant bacteria. In the control samples, *Corynebacteriaceae*, *Ruminococcacea*e were the predominant family.

**Fig 6:**
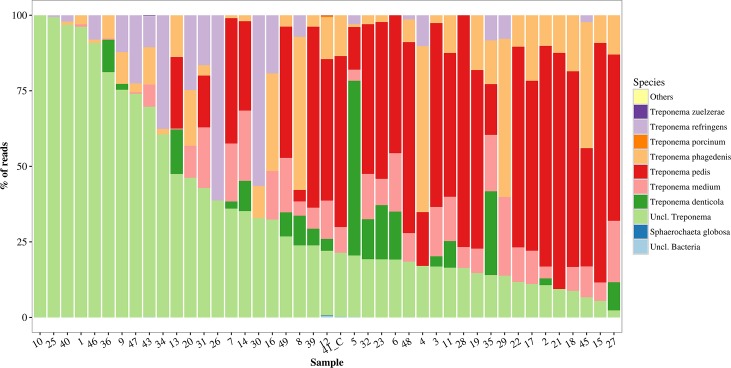
Relative abundance of *Treponema* phylotypes and other bacteria amplified by high-throughput sequencing in bovine digital dermatitis. Sequencing amplified the V3 and V4 hypervariable regions of 16S rRNA in 43 biopsy specimens of bovine digital dermatitis and 1 control sample from crossbreed dairy cows kept in pasture all year round. Control samples are identified by _C.

*Treponema* species identified by V3-V4 primers were *T*. *pedis*, *T*. *refringens*, *T*. *denticula*, *T*. *phagedenis*, *T*.*medium*, *T*. *porcinum* and *T*. *zuelzerae* (Fig 6). Other bacteria amplified by the V3-V4 primers were *Sphaerochaeta globosa*.

**Fig 7:**
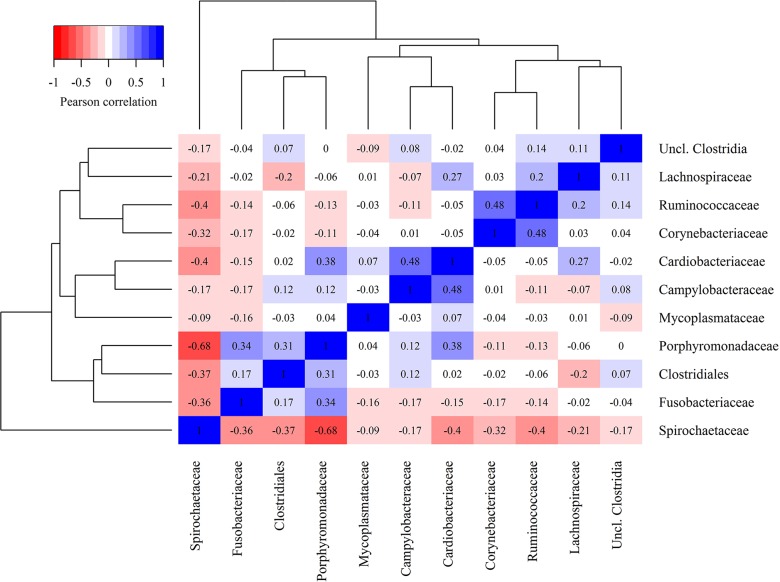
Heat map demonstrating the correlation of bacterial families across bovine digital dermatitis and control samples.

A correlation analysis of the families identified from the deep sequencing analysis is shown in Fig 7. In the heathy skin samples, the *Corynebacteriaceae* and *Ruminococcaceae* were moderately correlated (r^2^ = 0.48). The family *Cardiobacteriacea* which *D*. *nodosus* belongs to was moderately and positively correlated with *Campylobacteraceae* (r^2^ = 0.48) and *Porphyromonadaceae* (r^2^ = 0.38). The *Porphyromonadaceae* was also positive correlated with *Fusobacteriaceae* (r^2^ = 0.38). *Spirochaetaceae* was negatively correlated with almost all other bacterial families, showing a medium to low coefficient and a strong (r^2^ = -0.68) negative coefficient towards *Porphyromonadaceae*.

## Discussion

Although DD is found worldwide, attempts to study its etiology have been restricted to temperate climate regions where Holstein is the principal breed and most herds are housed in free stalls [3, 5, 7, 10, 21, 22]. In this study, we investigated samples from Brazilian grazing cattle by molecular methods to determine whether differences in environment, climate and geographical distance could reveal significant factors that determine the composition of the bacterial populations present in DD lesions.

In the DD lesions, *Treponema* was the dominant bacteria representing more than 50% of total reads in 52.3% of biopsy specimens. We identified eleven different *Treponema* strains belonging to the six major *Treponema* phylotypic clusters which have previously been described from DD lesions in other parts of the world; *T*. *phagedenis*-like, *T*. *refringens*-like, *T*. *denticola*/*pedis*-like, *T*. *medium*/*vicentii*-like, *T*. *porcinum*-like and *T*. *zuelzerae*-like [6, 7, 9, 21]. The most frequent and abundant types were *T*. *pedis*, *T*. *medium*, *T*. *phagedenis*, and *T*. *refringens*, which were also found in deeper parts of the lesions by FISH, much in accordance with other reports [6, 23]. Only one DD sample was negative for Treponema. This sample was only analyzed by FISH and in histopathological evaluation presented only mild alteration (score 1), characterized by epidermal hyperplasia, what may indicate that this lesion was in a final healing stage.

Besides the known DD pathogens, the phylogenetic analysis of the amplicons sequenced revealed a large number of *Treponema* reads which could only be classified to genus level. The possible reason is the existence of a larger number of not-yet classified *Treponema* species in this part of the world. However, is not possible to determine whether this unknown population constitutes an important contributor to DD development in this scenario, or that it is mainly composed of environmental treponemes without pathological importance.

Biopsy specimens from DD lesions presented lower bacterial diversity than control samples in the Shannon index. As can be seen in Fig 5, *Treponema* and *Porphyromonas* normally dominated the bacterial population in lesions specimens, while in control samples it was not possible to distinguish a predominant genus. This was described previously by Krull et al. (2014) [5] and Nielsen et al. (2016) [6], who also found *Treponema* and *Porphyromonas* to be the genera that differentiate control and DD samples most, together with *Mycoplasma* and *Fusobacterium*. One control sample which was taken from a macroscopically healthy skin 2 cm away from DD lesion, was positive for *Treponema*, *Porphyromonas* and *Fusobacterium*, similar to DD lesions. Additionally, FISH and histopathology analyses showed an increase in thickness of the epidermis with a high abundance of *T*. *refringens*, *T*. *medium* and *T*. *phagadenis*. The biopsy area was partially covered by hair. This may indicate that the DD lesion in that foot was expanding and microscopic changes were taking place, although there was no visible alteration on the surrounding area. A similar technique for collecting healthy skin samples was used by Zinicola et al. (2015) [7] with success.

In Brazil, a previous study was successful in demonstrating the groups *T*. *medium/T*. *vincentii-*like, *T*. *phagedenis-*like, and *T*. *denticola/T*. *putidum-*like in DD lesions using a nested-PCR method [14]. However, no information about the environment where the animals were kept is available, nor taxonomic origin of the other bacteria present. Pasture is advocated to be a less contaminated environment compared to confined facilities, and therefore generally presents lower hoof lesions and lameness prevalence [24]. Nonetheless, the visited farms presented a high prevalence of DD and all the main *Treponema* phylotypes and the majority of other bacteria frequently reported in DD could be found. This highlights the importance of DD worldwide, independent of housing conditions.

FISH analyses revealed a shift in bacterial population between preserved and degraded epidermis areas. In areas with mild epithelial damage, characterized by hyperkeratosis and acanthosis, the bacteria population was composed of sparse presence of spirochetes and *D*. *nodosus*. The *Treponema* population increased toward areas where the epidermis was more degraded, with partial loss of the epithelium and parakeratosis. The observed microbiota then shifted to a predominance of *P*. *levii* and *F*. *necrophorum*, and rare *Treponema* in areas with ulcers, hemorrhages, and exudate. The bacterial family correlation shown in Fig 7 corroborate with the spatial observation from FISH, indicating that *Treponema* and *P*. *levii* colonize different areas of the lesion. These finding are in accordance with previous work by our research group and others [3, 6, 9, 23].

We successfully found *D*. *nodosus* in DD lesions samples using 16S rRNA gene sequencing and also using FISH, as described before [9, 25]. *D*. *nodosus* is associated with several diseases in ruminants foot, and was advocated to participate in early lesions in bovine DD [9, 25]. In our results, *D*. *nodosus* was highly prevalent in FISH specimens, with almost 50% of positive samples. Although normally found superficially and in epidermal lesions, it could also be detected deeper, and besides that, it was observed in all DD developing stages, from the very beginning, to acute and chronic forms. These finding indicate the participation of *D*. *nodosus* in DD etiology, not only in beginning stages, but also in all the different macroscopic stages. In this context, one could speculate that *D*. *nodosus* has a role in the development and expansion of the lesion, together with *Treponema* species.

The spatial distribution of *P*. *levii*, despite its high prevalence, and *F*. *necrophorum*, suggest these species to be secondary invaders, and not the main etiological organisms. This is also suggested by other authors [5–7]. However, in a recent study using gene expression to clarify DD etiology, *P*. *levii* was indicated to participate in disease development, while the involvement of *F*. *necrophorum* was not sustained [26]. Therefore, although our results do not support the participation of either of these two bacteria in DD progression, further studies are warranted.

Among the other bacteria found in DD lesions, *Mycoplasma* has been advocated to participate in the transition between early phases to active lesions [5, 6], but this study had no observation supporting this theory. Similarly, we did not identify the bacteria *Guggenheimella bovis* nor *Candidatus Amoebophilus asiaticus*, which were described to be part of DD microbiota in studies by Schlafer et al. (2008) [27] and Zinicola et al. (2015) [7], respectively. *Helcococcu*s, *Campylobacter*, *Catonella* and an unclassified bacterium from class *Clostridia* were abundant bacteria present in biopsy specimens in this study. However, neither of these bacteria seem to be essential to DD development. In this context, it seems very likely that, except for *Treponema* spp., the bacterial species involved in DD vary enormously, but their importance for disease development is yet to be clarified.

## Conclusions

We have demonstrated that the dominant microbiota of DD lesions from all year grazing cattle are similar to previous studies from all year free stall housing, and possess all the six main *Treponema* phylotypes described previously. Furthermore, our results corroborate with the theory that DD is a polymicrobial disease and *Treponema* is the main bacteria responsible for disease development. In this case, *T*. *pedis*, *T*. *medium*, *T*. *phagedenis*, and *T*. *refringens* were the most abundant treponemes found. Among the other bacteria present in lesions, *D*. *nodosus* seems to play an important role in the development and expansion of the lesion, possibly acting together with *Treponema*. Additionally, we could not find any evidence of the participation of *P*. *levii*, despite their great abundance, and *F*. *necrophorum*, or any other bacteria, in disease development.

## Supporting information

S1 Table Names and sequences of 16S rRNA-targeting oligonucleotide probes and primers used in this study. (DOCX)
