# Supplementary material for: Pathology and bacteria related to digital dermatitis in dairy cattle in all year round grazing system in Brazil
Source: PLoS One. 2018 Mar 7;13(3):e0193870. doi: 10.1371/journal.pone.0193870 (PMC5841792; doi:10.1371/journal.pone.0193870)
Supplement: S1 Table — (DOCX) [file pone.0193870.s001.docx]

**Table S1. Names and sequences of 16S rRNA-targeting oligonucleotide probes and primers used in this study.**

| Probe name (no.) | Sequence | Target | Reference |
| --- | --- | --- | --- |
| S-S-Trep-Dig3-432 | 5’-CAT CCC AGT ATC ATT CCC-3’ | Uncultured Treponema PT1 | [1] |
| S-S-Trep-T16-432 | 5’-CAT CTC ACA GGC ATT CCC-3’ | *T. denticula* | [1] |
| S-S-Trep-I:B:C7-432 | 5’-CAT CAG ATG AGC ATT CCC-3’ | *T.medium* | [1] |
| S-S-TrepGenus-725 | 5’-CAG AAA CYC GCC TTC GCC-3’ | *Treponema* | [1] |
| S-S-F.necro-183-Cy3 ( | 5’-GAT TCC TCC ATG CGA AAA-3’ | *Fusobacterium necrophorum* | [2] |
| Eub-338 | 5’-GCT GCC TCC CGT AGG AGT’-3 | Bacteria | [3] |
| S-D.nodosus-443 | 5’-CAT GCA CCG TTC TTC ACT’-3 | *D. nodosus* | [4] |
| P. levii-443 | 5’-TACCTACGTTTACTCGCC-3’ | *P. levii* | [5] |
| PT12-216 | 5'-CG AGC CCA TCT TTA GGC GAA G-3' | Uncultured *Treponema* PT12 (PN20) | [4] |
| PT13 | 5'-GT AGC TCC TTT CCC TTC ACC TTA A- 3' | Uncultured *Treponema* PT13 | [4] |
| T. pedis | 5'-AG AGT CCT CAA CCT TTA CGT GTT-3' | *T. pedis* | [4] |
| T. refringens | 5'-GC TCC CTT TCC TTA CAT GAT-3' | *T. refringens* | [4] |
| S-S-Trep-Dig4-432 | 5’-CAT CTC AGT GTC ATT CCC-3’ | Uncultured *Treponema* PT3 | [1] |
| Primer name | Sequence | Target | Reference |
| V1-V2 forward | AGAGTTTGATCCTGGCTCAG | Bacteria | [6] |
| V1-V2 reverse | CTGCTGCCTYCCGTA | Bacteria | [6] |
| V3-V4 forward | GGGAGGCAGCAGCTAAGAA | *Treponema* spp. | [1] |
| V3-V4 reverse | ATCTACAGATTCCACCCCTA | *Treponema* spp. | [1] |

**References**

1. Klitgaard K, Boye M, Capion N, Jensen TK. Evidence of multiple *Treponema* phylotypes involved in bovine digital dermatitis as shown by 16S rDNA analysis and fluorescent in situ hybridisation. J Clin Microbiol. 2008; 46:3012-3020.
2. Boye M Aalbaek B and Agerholm JS. *Fusobacterium necrophorum* determined as abortifacient in sheep by laser capture microdissection and fluorescence in situ hybridization. Mol Cell Probes. 2006; 20:330-336.
3. Amann RI, Krumholz L, Stahl DA. Fluorescent-oligonucleotide probing of whole cells for determinative, phylogenetic, and environmental studies in microbiology. J Bacteriol. 1990; 172:762-770.
4. Rasmussen M, Capion N, Klitgaard K, Rogdo T, Fjeldaas T, Boye M, Jensen TK. Bovine digital dermatitis: possible pathogenic consortium consisting of *Dichelobacter nodosus* and multiple *Treponema* species. Vet Microbiol. 2012; 160:151-161.
5. Nielsen MW, Strube ML, Isbrand A, Al-Medrasi WDHM, Boye M, Jensen TK, Klitgaard K. Potential bacterial core species associated with digital dermatitis in cattle herds identified by molecular profiling of interdigital skin samples. Vet Microbiol. 2016; 186:139–149.
6. Wilmotte A Van der AG and De WR. Structure of the 16S ribosomal RNA of the thermophilic cyanobacterium *Chlorogloeopsis* HTF (*'Mastigocladus laminosus* HTF') strain PCC7518, and phylogenetic analysis. FEBS Lett. 1993; 317:96-100.
